# Accelerated Computing in Magnetic Resonance Imaging: Real-Time Imaging Using Nonlinear Inverse Reconstruction

**DOI:** 10.1155/2017/3527269

**Published:** 2017-12-31

**Authors:** Sebastian Schaetz, Dirk Voit, Jens Frahm, Martin Uecker

**Affiliations:** ^1^Biomedizinische NMR Forschungs GmbH, Max Planck Institute for Biophysical Chemistry, Göttingen, Germany; ^2^DZHK (German Centre for Cardiovascular Research), Partner Site Göttingen, Göttingen, Germany; ^3^Department of Diagnostic and Interventional Radiology, University Medical Center Göttingen, Göttingen, Germany

## Abstract

**Purpose:**

To develop generic optimization strategies for image reconstruction using graphical processing units (GPUs) in magnetic resonance imaging (MRI) and to exemplarily report on our experience with a highly accelerated implementation of the nonlinear inversion (NLINV) algorithm for dynamic MRI with high frame rates.

**Methods:**

The NLINV algorithm is optimized and ported to run on a multi-GPU single-node server. The algorithm is mapped to multiple GPUs by decomposing the data domain along the channel dimension. Furthermore, the algorithm is decomposed along the temporal domain by relaxing a temporal regularization constraint, allowing the algorithm to work on multiple frames in parallel. Finally, an autotuning method is presented that is capable of combining different decomposition variants to achieve optimal algorithm performance in different imaging scenarios.

**Results:**

The algorithm is successfully ported to a multi-GPU system and allows online image reconstruction with high frame rates. Real-time reconstruction with low latency and frame rates up to 30 frames per second is demonstrated.

**Conclusion:**

Novel parallel decomposition methods are presented which are applicable to many iterative algorithms for dynamic MRI. Using these methods to parallelize the NLINV algorithm on multiple GPUs, it is possible to achieve online image reconstruction with high frame rates.

## 1. Introduction

Accelerators such as graphical processing units (GPUs) or other multicore vector coprocessors are well-suited for achieving fast algorithm run times when applied to reconstruction problems in medical imaging [1] including computed tomography [2], positron emission tomography [3], and ultrasound [4]. This is because respective algorithms usually apply a massive number of the same independent operations on pixels, voxels, bins, or sampling points. The situation maps well to many-core vector coprocessors and their large number of wide floating-point units that are capable of executing operations on wide vectors in parallel. The memory bandwidth of accelerators is roughly one order of magnitude greater than that of central processing units; they are optimized for parallel throughput instead of latency of a single instruction stream.

Recent advances in magnetic resonance imaging (MRI) pose new challenges to the implementations of associated algorithms in order to keep data acquisition and reconstruction times on par. While acquisition times decrease dramatically as in real-time MRI, at the same time the data grows in size when using up to 64 or even 128 independent receiver channels on modern MRI systems. In addition, new modalities such as model-based reconstructions for (dynamic) parametric mapping increase the computational complexity of reconstruction algorithms because they in general involve iterative solutions to nonlinear inverse problems. As a consequence, accelerators are increasingly used to overcome these challenges [5–13].

**Figure 1:**
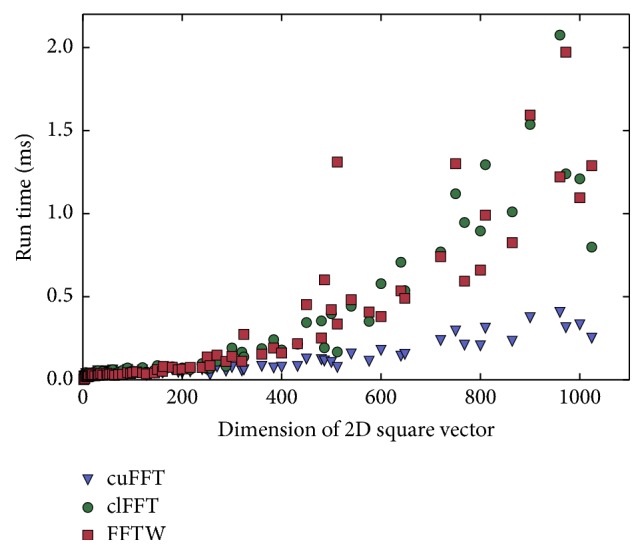
Run time (i.e., minimal wall-clock time in ms) measurement of a single-precision 2D squared complex-to-complex out-of-place Fourier transform of FFTW, clFFT, and cuFFT libraries. clFFT and cuFFT benchmarks were obtained for NVIDIA GeForce Titan Black; the FFTW benchmark was obtained for Intel Xeon E5-2650 utilizing 8 threads. FFTW is initialized with FFTW_MEASURE. The clFFT library has only limited support for mixed radix FFTs and achieves good performance for power of 2 FFTs only.

**Figure 2:**
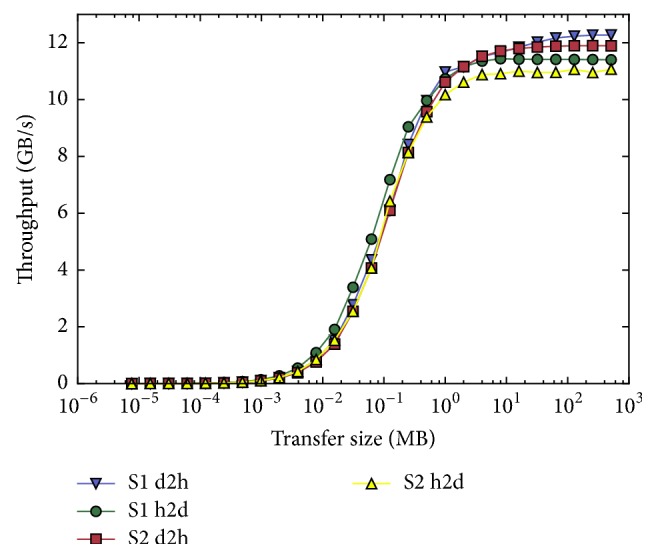
Throughput of Supermicro (S1) and Tyan (S2) 8x PCIe 3.0 systems: device-to-host and host-to-device transfer of nonpageable (pinned) memory with junk sizes between 8 bytes and 1 GB.

Accelerators outperform main processors (CPUs) in all important operations during MRI reconstruction including interpolation [14], filtering [15], and basic linear algebra [16]. The central operation of most MRI reconstruction algorithms is the Fourier transform.  Figure 1 shows the performance of the three FFT libraries cuFFT, clFFT, and FFTW [17]. The accelerator libraries cuFFT and clFFT outperform the CPU library FFTW. Consequently, accelerators outperform CPUs for MRI reconstruction, if the algorithm is based on the Fourier transform and if the ratio of computation to data transfer favours computation. Accelerator and CPU typically form distributed memory systems.  Figure 2 shows the memory transfer speed for two different PCIe 3.0 systems in dependence of the data transfer size. The amount of computation assigned to an accelerator and the transfer size have a huge impact on the overall performance of the algorithm, which needs to be taken into account when implementing accelerated algorithms for image reconstruction. Another limiting factor for the use of accelerators in MRI is the restricted amount of memory available. Nevertheless, the on-board memory of accelerators increased in recent years and the newest generation of accelerators is equipped with acceptable amounts of memory (compare Table 1).

In this paper, we summarize our experience in developing a low-latency online reconstruction system for real-time MRI over the last eight years. While some of the described techniques have already been reported previously, this article for the first time explains all technical aspects of the complete multi-GPU implementation of the advanced iterative reconstruction algorithm and adds further background information and analysis. Although we focus on the specific application of real-time MRI, many of the techniques developed for this project can be applied to similar tomographic reconstruction problems. In particular, we describe strategies for optimal choice of the grid size for a convolution-based nonuniform FFT, novel parallelization schemes using temporal and spatial decomposition, and automatic tuning of parameters.

## 2. Theory

### 2.1. Real-Time MRI

Diagnostic imaging in real time represents a most demanding acquisition and reconstruction problem for MRI. In principle, the data acquisition refers to the recording of a large number of different radiofrequency signals in the time domain which are spatially encoded with the use of magnetic field gradients. The resulting dataset represents the* k*-space (or Fourier space) of the image. MRI acquisition times are determined by the number of different encodings needed for high-quality image reconstruction multiplied by the time required for recording a single MRI signal, that is, the so-called repetition time TR. While TR values could efficiently be reduced from seconds to milliseconds by the invention of low-flip angle gradient-echo MRI sequences, for example, see [18] for an early dynamic application, a further speed-up by reducing the number of encodings was limited by the properties of the Fourier transform which for insufficient coverage of* k*-space causes image blurring and/or aliasing artifacts. Reliable and robust improvements in acquisition speed by typically a factor of two were first achieved when parallel MRI techniques [19, 20] were introduced. These methods compensate for the loss of spatial information due to data undersampling by simultaneously acquiring multiple datasets with different receiver coils. In fact, when such multicoil arrangements are positioned around the desired field-of-view, for example, a head or thorax, each coil provides a dataset with a unique spatial sensitivity profile and thus complementary information. This redundancy may be exploited to recover the image from moderately undersampled* k*-space data and thus accelerate the scan.

Parallel MRI indeed was the first concept which changed the MRI reconstruction from a two-dimensional FFT to the solution of an inverse problem. To keep mathematics simple and computations fast, however, commercially available implementations turned the true nonlinear inverse problem, which emerges because the signal model contains the product of the desired complex image and all coil sensitivity profiles (i.e., complex images in themselves), into a linear inverse problem. This is accomplished by first determining the coil sensitivities with the use of a prescan or by performing a low-resolution Fourier transform reconstruction of acquisitions with full sampling in the center of* k*-space and undersampling only in outer regions.

**Figure 3:**
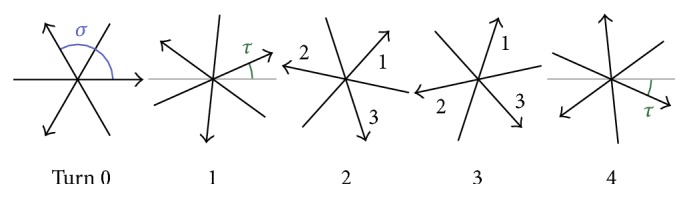
Schematic acquisition scheme for real-time MRI with *U* = 5 different sets of spokes, *K* = 3, *σ* = 2*π*/*K*, and *τ* = 2*π*/(*KU*).

Recent advances towards real-time MRI with so far unsurpassed spatiotemporal resolution and image quality [21] were therefore only possible by combining suitable acquisition techniques with an adequate image reconstruction. Crucial elements include the use of (i) rapid low-flip angle gradient-echo sequences, (ii) spatial encodings with radial rather than Cartesian trajectories, (iii) different (i.e., complementary) sets of spokes in successive frames of a dynamic acquisition (see Figure 3) [22], (iv) extreme data undersampling for each frame, (v) an image reconstruction algorithm (NLINV) that solves the nonlinear inverse problem (see below), and (vi) temporal regularization to the preceding frame which constrains the ill-conditioned numerical problem with physically plausible a priori knowledge. Real-time MRI with NLINV reconstruction achieves a temporal resolution of 10 to 40 ms per frame (i.e., 25 to 100 frames per second) depending on the actual application (e.g., anatomic imaging at different spatial resolutions or quantitative blood flow studies); for a recent review of cardiovascular applications, see [23].

The spatial encoding scheme is comprised of *U* different sets (turns) of *K* spokes. All *U* sets of spokes taken together cover the *k*-space uniformly. Figure 3 illustrates the real-time MRI encoding scheme.

### 2.2. Image Reconstruction with NLINV

If the receive coil sensitivities in parallel MRI are known, the image recovery emerges as a linear inverse problem which can efficiently be solved using iterative methods. In practice, however, static sensitivities are obtained through extrapolation and, even more importantly, in a dynamic in vivo setting they change due to coupling with the conductive tissue: this situation applies to a human subject during any type of movement (e.g., breathing or cardiac-related processes) or when dynamically scanning different planes and orientations (e.g., during real-time monitoring of minimally invasive procedures).

In such situations, extrapolating static sensitivities is not sufficient. The sensitivities have to be jointly estimated together with the image, yielding an inverse reconstruction problem that is nonlinear and ill-posed. For real-time MRI, both the dynamic changes during a physiologic process and the need for extremely undersampled datasets (e.g., by a factor of 20) inevitably lead to a nonlinear inverse problem.

A powerful solution to this problem is the regularized nonlinear inverse reconstruction algorithm [24]. NLINV formulates the image reconstruction as a nonlinear least-squares problem

(1) $$\begin{array}{c} \underset{x}{\mathrm{argmin}}\underset{\text{data fidelity}}{\underset{︸}{{\left\|Fx-y\right\|}_{2}^{2}}}+\underset{\text{regularization}}{\underset{︸}{\alpha {\left\|W\left(x-x_{\text{prev}}\right)\right\|}_{2}^{2}}}. \end{array}$$

The nonlinear forward operator *F* = *ℱ*∘*C* maps the combined vector *x* of the unknown complex-valued image *ρ* and all coil sensitivities *c*_*j*_ (i.e., complex-valued images in themselves) to the data. *C* multiplies the image with the sensitivities to obtain individual coil images *c*_*j*_*ρ* and *ℱ* then predicts the* k*-space data using a nonuniform Fourier transform for all coil elements *j* = 1,…, *J* (this nonuniform FFT is implemented here with a uniform FFT and convolution with the PSF [25], which is especially advantageous for implementation on a GPU [9]). The data fidelity term quantifies the difference between this predicted data and the measured data in the least-squares sense, while the additional regularization term addresses the ill-posedness of the reconstruction problem. Here, *W* is a weighting matrix in the Fourier domain which characterizes the spatial smoothness of the coil sensitivities and does not change the image component of *x*. For dynamic imaging, temporal regularization to the immediately preceding frame *x*_prev_, which exploits the temporal continuity of a human movement or physiologic process, allows for a remarkably high degree of data undersampling or image acceleration [21].

In the following, the main steps of an efficient numerical implementation are described; for further details, see [9, 21]. First, a change of variables $\hat{x}=Wx$ and $\hat{F}=F\circ W^{-1}$ is done to improve the conditioning of the reconstruction problem. Starting from an initial guess *x*_0_, the numerical problem is then solved using the iteratively regularized Gauss-Newton method (IRGNM), which uses the following linear update rule:

(2) DF^x^nHDF^x^n+αnIx^n+1−x^n=DF^x^nHy−F^x^n−αnx^n−x^prev.

Here, $D{\hat{F}}_{{\hat{x}}_{n}}$ denotes the derivative of $\hat{F}$ at ${\hat{x}}_{n}$ and $D{\hat{F}}^{H}$ its adjoint. In each of the *M* Newton steps, this linear system of equations is solved using the method of conjugate gradients (CG). The main operation in a matrix-free implementation is the repeated application of the operator

(3) $$\begin{array}{c} D{\hat{F}}_{{\hat{x}}_{n}}^{H}D{\hat{F}}_{{\hat{x}}_{n}}:\delta x⟼\left(\begin{array}{c} \overset{J}{\underset{j=1}{\sum }}c_{j}^{⋆}t_{j}\\ W_{b}^{-H}\rho^{⋆}t_{1}\\ \vdots \\ W_{b}^{-H}\rho^{⋆}t_{J} \end{array}\right) \end{array}$$

with

(4) $$\begin{array}{c} t_{j}≔F_{b}^{H}F_{b}\left\{\;c_{j}\delta \rho +\rho W_{b}^{-1}\delta {\hat{c}}_{j}\;\right\}. \end{array}$$

**Figure 4:**
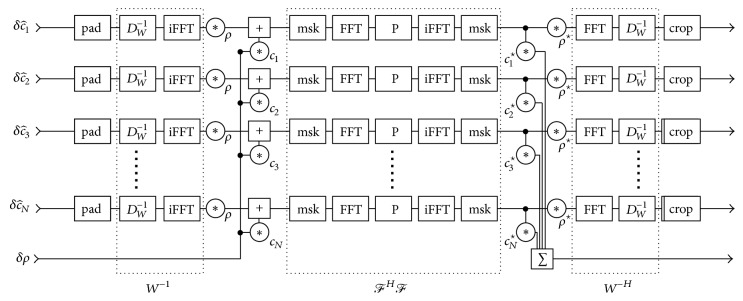
Flowchart of the operator $D{\hat{F}}_{{\hat{x}}_{n}}^{H}D{\hat{F}}_{{\hat{x}}_{n}}$. The implementation of the weighting matrix *W*^−1^ and its adjoint *W*^−*H*^ applies a diagonal weighting matrix *D*_*W*_^−1^ and a forward or inverse fast Fourier transform (FFT, iFFT) for each channel. The derivative of the multiplication of the image *ρ* and the coil sensitivities *c*_*j*_ and its adjoint are composed of pointwise multiplication (*∗*), addition (+), and summation (∑) operations. The combination of the nonuniform Fourier transform and its adjoint *ℱ*^*H*^*ℱ* is implemented as a convolution with the point-spread function, consisting of a mask (msk) restricting the oversampled grid to the field of view, application of fast Fourier transform and its inverse, and a pointwise multiplication with the convolution kernel (P). Padding (pad) and cropping (crop) operations are used to reduce computational cost in the overall iterative algorithm.

The star ⋆ means complex conjugation. *ℱ*_*b*_ and *W*_*b*_ are the blocks of the block-diagonal operations *ℱ* and *W* which operate on a single channel. Because the nonuniform Fourier transform is paired with its adjoint, it can be implemented efficiently as a truncated convolution with a point-spread function using two applications of an FFT algorithm on a twofold oversampled grid [25]. *W* is implemented using one FFT followed by the application of a diagonal weighting matrix. Thus, a single iteration requires 4 applications of an FFT algorithm per channel, several pixelwise complex multiplications and additions, and one pixel-by-pixel reduction across all channels (∑_*j*=1_^*J*^). A flowchart can be found in Figure 4.

The NLINV algorithm is an iterative algorithm consisting of operators *F*, $D\hat{F}$, and $D{\hat{F}}^{H}$ as well as the conjugate-gradient method. Depending on the imaging scenario, the number of Newton steps is fixed to 6 to 10. *F* is only computed once per Newton step. $D\hat{F}$ and $D{\hat{F}}^{H}$ are applied in each conjugate-gradient iteration. Each operator applies a 2D FFT twice on per-channel data. *F* computes a scalar product once; the conjugate gradient method contains two scalar products over all channel data. Furthermore each operator applies between 4 and 6 elementwise operations and $D{\hat{F}}^{H}$ contains a summation over channel data. The performance of the Fourier transform dominates the run time of a single inner-loop iteration of the algorithm and it has to be applied 4 times in each inner loop.

If 10 channels are assumed, 6 Newton steps are computed (yielding roughly 50 conjugate-gradient iterations); 10*∗*4*∗*50 = 2000 2D Fourier transformations must be applied to compute a single image. If data is acquired at a frame rate of 30 fps, the reconstruction system must be able to apply 60000 2D Fourier transforms per second.

## 3. Optimization Methods and Results

The following section outlines several steps undertaken to move a prototype implementation of the NLINV algorithm to a highly accelerated implementation for use in an online reconstruction pipeline. The original implementation in C utilizes the CUDA framework and is capable of reconstructing at speeds of 1 to 5 fps on a single GPU. A typical clinical scenario in real-time MRI is cardiac imaging where a rate of 30 per second is necessary. To incorporate real-time heart examination in a clinical work-flow, the image reconstruction algorithm must perform accordingly.

The optimization techniques are classified into two categories. The first category encompasses platform independent optimization procedures that reduce the overall computational cost of the algorithm by reducing vector sizes while maintaining image quality. In the second category, the algorithm is modified to take advantage of the multi-GPU computer platform by channel decomposition, temporal decomposition, and tuning of the two techniques to yield best performance for a given imaging scenario.

All benchmark results show the minimum wall-clock time of a number of runs. For microbenchmarks, hundreds of runs were performed. For full reconstruction benchmarks, tens of runs were performed. The speed-up *S* is defined as the quotient of the old wall-clock time *t*_old_ over the new time *t*_new_

(5) $$\begin{array}{c} S=\frac{t_{old}}{t_{new}} \end{array}$$

and parallel efficiency *E* is defined as

(6) $$\begin{array}{c} E=\frac{S_{p}}{p} \end{array}$$

with *S*_*p*_ representing the speed-up achieved when using *p* accelerators over 1. Perfect efficiency is achieved when *S*_*p*_ = *p* or *E* = 1; sublinear speed-up is measured if *S*_*p*_ < *p* and *E* < 1.

If not stated otherwise, all benchmarks were obtained on a Supermicro SuperServer 4027GR-TR system with PCIe 3.0, 2x Intel Xeon Ivy Bridge-EP E5-2650 main processors, 8x NVIDIA Titan Black (Kepler GK110) accelerators with 6 GB graphics memory each, and 128 GB main system memory. Custom GPU power cables were fabricated for the Supermicro system to support consumer-grade accelerators. The CUDA run time environment version was 6.5 with GPU driver version 346.46. The operating system used was Ubuntu 14.04. The combined single-precision performance of the eight GPUs in this system approaches 41 TFLOPS and the combined memory bandwidth is 2688 Gb/s. For a comparison of the performances of some accelerators available at the time of writing of the article, see Table 1.

### 3.1. Real-Time Pipeline

The real-time NLINV reconstruction algorithm is part of a larger signal processing pipeline that can be decomposed into several stages:

- Datasource: reading of input data into memory
- Preprocessing: interpolating data acquired with a non-Cartesian trajectory onto a rectangular grid, correcting for gradient delays as well as performing the channel compression; this stage contains a calibration phase that calculates the channel compression transform matrix and estimates the gradient delay
- Reconstruction: the NLINV algorithm, reconstructing each image
- Postprocessing: cropping of the image to the measured field of view, calculation of phase-difference image (in case of phase-contrast flow MRI), and applying temporal and spatial filters
- Datasink: writing of resulting image to output.

Real-time MRI datasets with high temporal resolution typically contain several hundreds of frames even when covering only a few seconds of imaging. For this reason, it is beneficial to parallelize the pipeline at the level of these functional stages: each stage can work on a different frame, or, in other words, different frames are processed by different stages of the pipeline in parallel. The implementation of such a pipeline follows the actor model [26] where each pipeline stage is one (or multiple, see temporal decomposition in Section 3.3) actor, receiving input data from the previous stage as message and passing results to the following stage as a different message. The first actor and the last actor only produce (datasource) and receive (datasink) messages, respectively.

**Figure 5:**
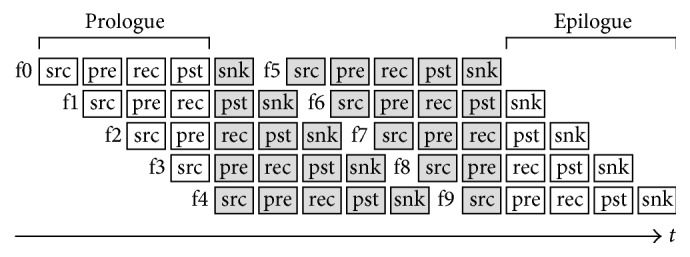
The NLINV reconstruction pipeline consists of 5 stages: datasource (src), preprocessing (pre), reconstruction (rec), postprocessing (pst) and datasink (snk). New frames (f0 through f9) enter the pipeline through the datasource and leave the pipeline through the datasink. A filled pipeline processes 5 frames at the same time by using 5 threads, one for each pipeline stage.

Figure 5 shows the NLINV pipeline for reconstructing 10 frames. The pipeline has a prologue and an epilogue of 4 frames (number of pipeline stages minus one) where full parallel reconstruction is not possible because the pipeline is filling up or emptying.

### 3.2. Computational Cost Reduction

**Figure 6:**
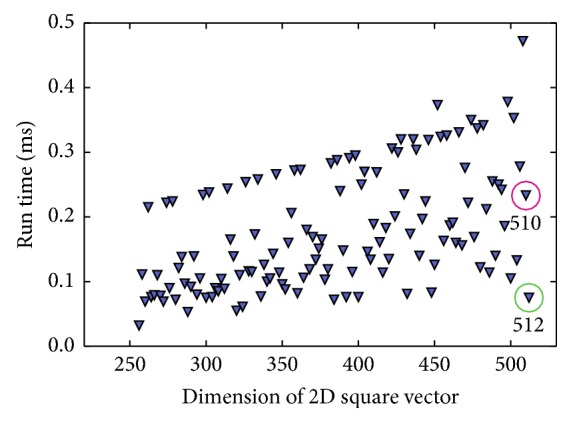
Run time in ms of a single-precision 2D complex-to-complex out-of-place squared Fourier transform calculated using cuFFT. The plot shows run times as a function of vector size and highlights the run time difference between a 510^2^ vector (red) and 512^2^ vector (green). The plotted data ranges from 256^2^ to 512^2^.

The most compute-intensive part of the NLINV algorithm is the application of the Fourier transform. NLINV applies the Fourier transform multiple times in each iteration. Fast execution of the Fourier transform has thus a major impact on the overall run time of the algorithm. The FFT library cuFFT [27] of the hardware vendor is used. Plotting the performance of the FFT libraries against the input vector size does not yield a linear function, but shows significant fluctuation (compare Figure 6). The algorithm performs better for vector sizes which are factorizable by small prime numbers, with the smallest prime numbers yielding the best results.

The grid oversampling ratio defines the ratio of grid sample locations *G* over the side length of the output image *N*. The ratio between *N* and *G* includes an inherent factor of 2 that exists as the convolution with the point-spread function requires twofold oversampling. An additional factor *γ* can be set to values between 1 and 2 to prevent aliasing artifacts for situations where the operator chooses a field of view smaller than the object size. The relationship of *N* to *G* is thus defined as

(7) $$\begin{array}{c} G=2\gamma N. \end{array}$$

In the following, only *γ* is specified. It is found that considering the constraints of the FFT algorithm when choosing *γ* can yield calculation speed-ups. A lookup table is generated that maps grid size to FFT performance by benchmarking the Fourier transform algorithm for all relevant input sizes. This lookup table is generated for different accelerator generations as well as for new versions of the FFT library. The grid oversampling ratio is adjusted according to the highest measured FFT performance with a minimum oversampling ratio of *γ* ≥ 1.4.  Table 2 shows the speed-up when comparing to a fixed oversampling ratio of 1.5. Figure 6 is a graphical representation of the lookup table that is used to select optimal grid sizes. It highlights the run time difference between two very similar vector sizes of 510^2^  (2*∗*3*∗*5*∗*17) and 512^2^  (2^9^).

**Figure 7:**
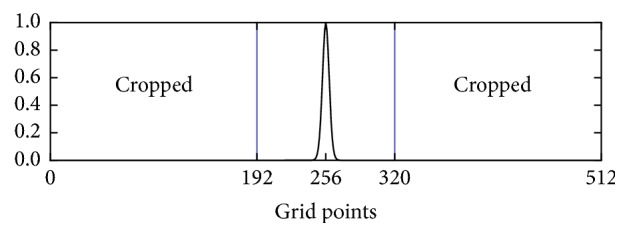
The* k*-space weight function (1 + 880|*k*|^2^)^16^ with −0.5 < *k*_*x*_, *k*_*y*_ < 0.5 is plotted in 1D for a grid size of 512^2^ with cropping to 25% of the original. The lowest frequency is at the center.

The NLINV algorithm estimates not only the spin-density map *ρ*, but also the coil sensitivity profiles *c*_*j*_. The regularization term added to the Gauss-Newton solver constrains the *c*_*j*_ coil sensitivity profiles to a few low-frequency components. It is thus possible to not store the entire vector *c*_*j*_, but to reduce its size to (1/4)^2^ of the original. The number of grid sample locations *G* is thus reduced to *G*_*c*_ = ⌊(1/4)*G*⌋ for the coil sensitivity profiles *c*_*j*_. Whenever the original size is required, the vector is padded with zeroes.  Figure 7 shows a typical weighting function in 1D as applied to *c*_*j*_ and the cut-off that is not stored after this optimization. This saves computing time as functions applied on *c*_*j*_ have to compute less. For this technique to yield a speed-up, the computation time saved must exceed the time required to crop and pad the vectors.  Table 3 shows the speed-up achieved by this optimization for various data sizes.

### 3.3. High-Level Parallelization with Autotuning

In parallel MRI, the reconstruction problem can be partitioned across the domain of multiple receive channels used for signal reception [10]. The measured data *y*_*j*_, corresponding coil sensitivity maps *c*_*j*_, and associated intermediate variables can be assigned to different accelerators *A*. The image *ρ* has to be duplicated on all accelerators. The sum ∑_*j*_^*J*^*c*_*j*_^⋆^*t*_*j*_ is partitioned across accelerators according to

(8) $$\begin{array}{c} \overset{J}{\underset{j}{\sum }}c_{j}^{⋆}t_{j}=\overset{A}{\underset{a=1}{\sum }}\overset{}{\underset{j\in J_{a}}{\sum }}c_{j}^{⋆}t_{j}, \end{array}$$

where *J*_*a*_ is the subset of channels assigned to accelerator *a*. This summation amounts to an all-reduce operation, since all accelerators require the computed updates to *ρ*. An alternative decomposition would require communication between GPUs within the FFT operation which is not feasible for the typical vector dimensions in this problem. The resulting speed-up from this parallel decomposition originates from the fact that the FFT of all channels can be computed in parallel. With only one accelerator, all *J* Fourier transforms have to be computed by a single accelerator with a batch size equal to *J*. A batched FFT computes multiple FFTs of the same size and type on multiple blocks of memory either in parallel or in a sequential manner. With multiple accelerators, *J* is divided by the number of accelerators *A*. This is illustrated in Table 4 depicting the computation time of Fourier transforms of 10 2D vectors of various side lengths *N* for different numbers of GPUs.

The parallel efficiency of distributing a batched FFT across devices decreases from two to three and four accelerators, because an accelerator can compute multiple 2D FFTs at the same time. In addition, the NLINV implementation uses 10 compressed channels which cannot be divided by 3 or 4 with no remainder.

Furthermore, the speed-up is reduced by the communication overhead of calculating ∑_*a*=1_^*A*^ which increases with the number of accelerators.  Table 5 shows the reconstruction speed and speed-up for differently sized datasets and varying number of accelerators. To reduce the communication overhead, a peer-to-peer communication technique is employed that allows accelerators to directly access memory of neighbouring accelerators. This is only possible, if accelerators share a PCIe domain. The Supermicro system has 2 PCIe domains that each connect 4 accelerators. The maximum effective number of accelerators that different channels are assigned to is therefore 4.

Because the parallel efficiency of the channel decomposition is limited, investigations focused on decomposing the problem along the temporal domain which results in reconstructions of multiple frames at the same time. The standard formulation of the NLINV algorithm prohibits a problem decomposition along the temporal domain. Frame *n* must strictly follow frame *n* − 1 as *x*_*n*−1_ serves as starting and iterative regularization value for *x*_*n*_. While maintaining the necessary temporal order, a slight relaxation of the temporal regularization constraint allows for the reconstruction of multiple frames at the same time. The following scheme ensures that the difference in the results of in-order and out-of-order image reconstruction remains minimal. The first frame *n* = 0 is defined with *ρ* set to unity and *c*_*j*_ set to zero. The function *h* maps frame *n* for each *n* > 0 and Newton step *m* to initialization and regularizations values *x*_*h*(*n*, *m*)_:

(9) $$\begin{array}{c} h\left(\;n,m\;\right)=\left\{\begin{array}{cc} n-1 & :n\leq l\vee m=M-1\\ \left[n-o,n-1\right] & :n>l\wedge m<M-1. \end{array}\right. \end{array}$$

**Figure 8:**
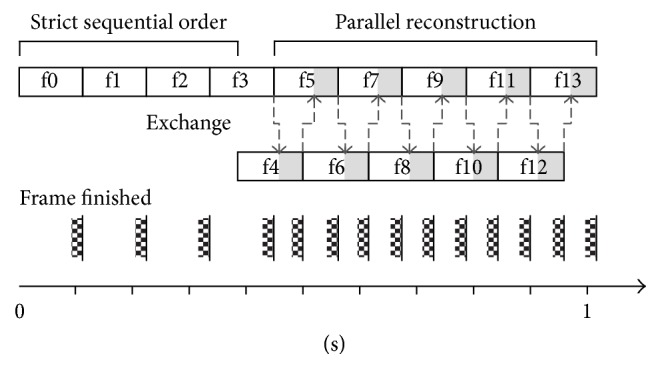
Temporal decomposition when reconstructing 14 frames. The first 4 frames are reconstructed in strict sequential order, while following images are reconstructed by two threads in parallel. Data is exchanged between threads for the last iteration (i.e., Newton step, grey segment) of each frame. This last iterative calculation takes the longest time as the CG algorithm requires more time to solve the system of equations. The frame rate is almost doubled by parallel reconstruction in this example.

Due to the complementary data acquisition pattern for sequential frames, the initial frames are of poor quality. The first *l* images of a series are thus reconstructed in a strict in-order sequence. This allows the algorithm to reach the best image quality in the shortest amount of time. From frame *l* + 1 on, frames may be reconstructed in parallel. Initialization and regularization values are chosen to be the most recent available frame within the range [*n* − *o*, *n* − 1]. The only exception is regularization values in the last Newton step *m* = *M* − 1, where *x*_*n*−1_ must be used for regularization. The algorithm thus waits for frame *n* − 1 to be computed before proceeding to the last Newton step (compare Figure 8). Experimental validation shows that the best match of in-order and out-of-order processing while maintaining a speed-up can be achieved by setting *l* to the number of turns and *o* to roughly half the number of turns in the interleaved sampling scheme.

Channel decomposition and temporal decomposition can be applied for different imaging situations in different ways. Depending on the acquisition and reconstruction parameters, the number of reconstruction threads *T* (temporal decomposition) and the number of accelerators per reconstruction thread *A* (channel decomposition) can be adjusted. A prerequisite to this method is that all (*T*, *A*) settings yield the same image quality. The parameters *P*_acqu_ and *P*_reco_ that have the most impact on image reconstruction speed include

- the imaging mode: single-slice anatomy, multislice anatomy, and phase-contrast flow,
- the data size which depends on the field of view and the chosen resolution,
- the number of frames acquired,
- the number of virtual channels used.

The number of parameters and the ever-evolving measurement protocols in a research setting make it difficult to come up with a model that maps the set of acquisition and reconstruction parameters to the optimal set of parallelization parameters (*P*_acqu_, *P*_reco_) → (*T*, *A*). Therefore, an autotuning mechanism is employed: The algorithm measures its own run time *R* and stores its performance along with all acquisition, reconstruction, and parallelization parameters in a database (*P*_acqu_, *P*_reco_) → (*T*, *A*) → *R*. For a given set of (*P*_acqu_ and *P*_reco_), the autotuning mechanism can select all recorded run times *R*, sort them, and select the set of parallelization parameters (*T*, *A*) that yields the best performance.

The autotuning has an optional learning mode to populate the database with varying parallelization parameters (*T*, *A*). If the learning mode is active, the algorithm searches the database for matching performance data and chooses parallelization arguments that do not yet exist in the database. The search space for (*T*, *A*) is limited; there are only 16 sets of arguments for the 8-fold GPU reconstruction system used here. The reason for this is the restriction of the channel decomposition stage to the size of the PCIe domain due to peer-to-peer memory access. If *A* = 1 then *T* = [1,2, 3,4, 5,6, 7,8], if *A* = 2, then *T* = [1,2, 3,4], and if *A* = [3,4] then *T* = [1,2].  Table 6 shows an excerpt from the autotuning database.

In a clinical setting, all relevant protocols may undergo a learning phase populating the database and covering the entire search space. This can be done in a setup phase to ensure optimal run times for all clinical scans. The algorithm is also capable of sorting acquisition and reconstruction parameters. This allows the autotuning mechanism to find good (*T*, *A*) parameters for new measurement protocols it has never seen before.

## 4. Discussion

This work describes the successful development of a highly parallelized implementation of the NLINV algorithm for real-time MRI where dynamic image series are reconstructed, displayed, and stored with little or almost no delay. The computationally demanding iterative algorithm for dynamic MRI was decomposed and mapped to massive parallel hardware. This implementation is deployed to several research groups and represents a cornerstone for the evaluation of the clinical relevance of a variety of real-time MRI applications, mainly in the field of cardiac MRI [23, 28], quantitative phase-contrast flow MRI [29, 30], and novel fields such as oropharyngeal functions during swallowing [31], speaking [32], and brass playing [33].

As examples, Supplementary Videos 1 and 2 show T1-weighted radial FLASH MRI acquisitions at 33.32 ms acquisition time (TR = 1.96 ms, 17 radial spokes covering* k*-space) of a human heart in a short-axis view and midsagittal tongue movements of an elite horn player, respectively. The cardiac example employed a 256 × 256 mm^3^ field of view, 1.6 mm in-plane resolution (6 mm slice thickness), and *N* = 160 data samples per spoke (i.e., grid size) which resulted in a slightly delayed reconstruction speed of about 22 fps versus 30 fps acquisition speed. In contrast, the horn study yielded real-time reconstruction speed of 30 fps because of a slightly smaller 192 × 192 mm^3^ field of view at 1.4 mm in-plane resolution (8 mm slice thickness) and only *N* = 136 samples per spoke.

Some of the optimization methods discussed here are specific to real-time MRI algorithms, but the majority of techniques translate well to other MRI applications or even to other medical imaging modalities. Functional decomposition can be employed in any signal or image processing pipeline that is built on multiple processing units like multicore CPUs, heterogeneous systems, or distributed systems comprised of FPGAs, digital signal processors, microprocessors, ASICs, and so on. Autotuning is another generic technique that can be useful in many other applications: if an algorithm implementation exposes parallelization parameters (number of threads, number of GPUs, distribution ratios, etc.), it can be tuned for each imaging scenario and each platform individually. The grid size optimization technique can also be universally employed: if an algorithm allows choosing the data size (within certain boundaries) due to regridding or interpolation, the grid size should be chosen such that the following processing steps exhibit optimal performance. This is valuable, if the following processing steps do not exhibit linear performance such as specific FFT implementations.

## 5. Conclusion

Novel parallel decomposition methods are presented which are applicable to many iterative algorithms for dynamic MRI. Using these methods to parallelize the NLINV algorithm on multiple GPUs, it is possible to achieve online image reconstruction with high frame rates. For suitable parameters choices, real-time reconstruction can be achieved.

## Figures and Tables

**Table 1:** Memory, memory bandwidth, and single-precision performance of high performance computing (HPC) and consumer accelerators (source: Wikipedia).

Vendor	Model	HPC	Memory	Bandwidth	Performance
NVIDIA	K40	yes	12 GB	288 GB/s	4.29 TFLOPS
NVIDIA	Titan Black	no	6 GB	336 GB/s	5.12 TFLOPS
AMD	FirePro W9100	yes	16 GB	320 GB/s	5.24 TFLOPS
AMD	Radeon R9 290X	no	6 GB	320 GB/s	5.63 TFLOPS
Intel	Xeon Phi 7120	yes	16 GB	352 GB/s	2.40 TFLOPS

**Table 2:** Reconstruction times in fps for various image sizes with side length *N* and fixed versus variable oversampling ratio *γ*. For some input sizes, 1.5 is the optimal value (*N* = 128, *N* = 144); for other sizes, the number of grid sample locations must be increased to yield a speed-up (*N* = 160, *N* = 170). For a third group of image sizes (*N* = 256), the optimal FFT size increases the size of the data to a degree that the speed-up is reduced or even nullified by additional overhead. For each test case, 200 frames were reconstructed using 1 accelerator.

*N*	Fixed *γ* = 1.5		Optimal *γ* ≥ 1.4			*S*
	*G*	fps	*γ*	*G*	fps	
128	384	8.1	1.5	384	8.3	1.02
144	432	2.5	1.5	432	2.5	1.00
160	480	4.4	1.51875	486	5.0	1.25
170	510	2.5	1.50588	512	5.8	2.32
256	768	2.4	1.53125	784	2.4	1.00

**Table 3:** Speed-up *S* by cropping the 2D vector of size length *N* of coil sensitivity profiles by a factor of (1/4)^2^.

*N*	*G*_*c*_ = *G*		Reduced *G*_*c*_ = ⌊(1/4)*G*⌋		*S*
	*G*_*c*_	fps	*G*_*c*_	fps	
128	384	18.1	96	25.8	1.43
144	432	10.1	108	13.0	1.29
160	486	11.9	121	17.9	1.50
170	512	13.4	128	20.1	1.50
256	784	6.1	196	9.7	1.59

**Table 4:** Computation time *t* in *μs* and parallel efficiency *E* of the Fourier transform of 10 2D vectors of side length *N* calculated using the cuFFT batched mode.

*N*	1 GPU	2 GPUs		3 GPUs		4 GPUs	
	*t*	*t*	*E*	*t*	*E*	*t*	*E*
384	429	230	0.93	189	0.76	152	0.71
432	532	280	0.95	228	0.78	178	0.75
486	730	387	0.94	320	0.76	251	0.73
512	555	288	0.96	233	0.79	179	0.78
784	1699	865	0.98	697	0.81	529	0.80

**Table 5:** Image reconstruction speed (fps), relative speed-up *S*_rel_, and overall speed-up *S*_ov_ for different numbers of GPUs and differently sized datasets.

*N*	1 GPU	2 GPUs		3 GPUs		4 GPUs		*S*_ov_
	fps	fps	*S*_rel_	fps	*S*_rel_	fps	*S*_rel_	
128	8.3	13.1	1.6	13.5	1.0	13.8	1.0	1.7
144	2.5	3.9	1.6	4.1	1.0	4.2	1.0	1.7
160	5.0	8.3	1.6	8.7	1.1	9.1	1.0	1.8
170	6.0	9.6	1.6	10.1	1.0	10.1	1.0	1.7
256	2.4	3.8	1.6	4.5	1.2	4.7	1.1	2.0

**Table 6:** Autotuning for reconstructing single-slice, dual-slice, and phase-contrast flow MRI acquisitions. *N* = 160 for all datasets results in a grid size of 486^2^.

Single-slice anatomic MRI						
	Worst configuration			Best configuration		
Frames	Threads	GPUs/thread	Fps	Threads	GPUs/thread	Fps
5	2	4	1.9	1	2	3.7
10	2	4	3.0	1	2	5.0
25	1	1	4.7	2	2	7.7
50	1	1	4.9	3	2	11.0
200	1	1	4.9	3	2	18.1

Dual-slice anatomic MRI						
	Worst configuration			Best configuration		
Frames	Threads	GPUs/thread	Fps	Threads	GPUs/thread	Fps

5	2	4	3.2	2	1	5.9
10	1	1	4.6	2	2	8.0
25	1	1	5.0	4	2	12.3
50	1	1	5.1	4	2	18.4
200	1	1	5.1	4	2	28.1

Phase-contrast flow MRI						
	Worst configuration			Best configuration		
Frames	Threads	GPUs/thread	Fps	Threads	GPUs/thread	Fps

5	2	4	1.6	2	1	2.6
10	1	1	1.8	2	2	3.6
25	1	1	1.9	4	2	5.8
50	1	1	1.9	4	2	7.5
200	1	1	1.9	4	2	10.7

## Supplementary Materials

Supplementary 1 Video S1: T1-weighted radial FLASH MRI acquisitions with NLINV reconstruction at 33.32 ms acquisition time (TR = 1.96 ms, 17 radial spokes) of a human heart in a short-axis view (256 × 256 mm^3^ field of view, 1.6 mm in-plane resolution, and 6 mm slice thickness).

Supplementary 2 Video S2: T1-weighted radial FLASH MRI acquisitions with NLINV reconstruction at 33.32 ms acquisition time (TR = 1.96 ms, 17 radial spokes) of midsagittal tongue movements of an elite horn player (192 × 192 mm^3^ field of view at 1.4 mm in-plane resolution, and 8 mm slice thickness).
